# Intraperitoneal Lavage with *Crocus sativus* Prevents Postoperative-Induced Peritoneal Adhesion in a Rat Model: Evidence from Animal and Cellular Studies

**DOI:** 10.1155/2021/5945101

**Published:** 2021-12-16

**Authors:** Pouria Rahmanian-Devin, Hassan Rakhshandeh, Vafa Baradaran Rahimi, Zahra Sanei-Far, Maede Hasanpour, Arghavan Memarzia, Mehrdad Iranshahi, Vahid Reza Askari

**Affiliations:** ^1^Department of Pharmaceutics, School of Pharmacy, Mashhad University of Medical Sciences, Mashhad, Iran; ^2^Pharmacological Research Center of Medicinal Plants, Mashhad University of Medical Sciences, Mashhad, Iran; ^3^Biotechnology Research Center, Pharmaceutical Technology Institute, Mashhad University of Medical Sciences, Mashhad, Iran; ^4^Department of Physiology, Faculty of Medicine, Mashhad University of Medical Sciences, Mashhad, Iran; ^5^Applied Biomedical Research Center, Mashhad University of Medical Sciences, Mashhad, Iran; ^6^Department of Pharmaceutical Sciences in Persian Medicine, School of Persian and Complementary Medicine, Mashhad University of Medical Sciences, Mashhad, Iran; ^7^Department of Persian Medicine, School of Persian and Complementary Medicine, Mashhad University of Medical Sciences, Mashhad, Iran

## Abstract

Postoperative peritoneal adhesions are considered the major complication following abdominal surgeries. The primary clinical complications of peritoneal adhesion are intestinal obstruction, infertility, pelvic pain, and postoperative mortality. In this study, regarding the anti-inflammatory and antioxidant activities of *Crocus sativus,* we aimed to evaluate the effects of *Crocus sativus* on the prevention of postsurgical-induced peritoneal adhesion. Male Wistar-Albino rats were used to investigate the preventive effects of *C. sativus* extract (0.5%, 0.25% and 0.125% *w*/*v*) against postsurgical-induced peritoneal adhesion compared to pirfenidone (PFD, 7.5% *w*/*v*). We also investigated the protective effects of PFD (100 *μ*g/ml) and *C. sativus* extract (100, 200, and 400 *μ*g/ml) in TGF-*β*1-induced fibrotic macrophage polarization. The levels of cell proliferation and oxidative, antioxidative, inflammatory and anti-inflammatory, fibrosis, and angiogenesis biomarkers were evaluated both *in vivo* and *in vitro* models. *C. sativus* extract ameliorates postoperational-induced peritoneal adhesion development by attenuating oxidative stress [malondialdehyde (MDA)]; inflammatory mediators [interleukin- (IL-) 6, tumour necrosis factor- (TNF-) *α*, and prostaglandin E_2_ (PGE_2_)]; fibrosis [transforming growth factor- (TGF-) *β*1, IL-4, and plasminogen activator inhibitor (PAI)]; and angiogenesis [vascular endothelial growth factor (VEGF)] markers, while propagating antioxidant [glutathione (GSH)], anti-inflammatory (IL-10), and fibrinolytic [tissue plasminogen activator (tPA)] markers and tPA/PAI ratio. In a cellular model, we revealed that the extract, without any toxicity, regulated the levels of cell proliferation and inflammatory (TNF-*α*), angiogenesis (VEGF), anti-inflammatory (IL-10), M1 [inducible nitric oxide synthase (iNOS)] and M2 [arginase-1 (Arg 1)] biomarkers, and iNOS/Arg-1 ratio towards antifibrotic M1 phenotype of macrophage, in a concentration-dependent manner. Taken together, the current study indicated that *C. sativus* reduces peritoneal adhesion formation by modulating the macrophage polarization from M2 towards M1 cells.

## 1. Introduction

Postoperative peritoneal adhesions are considered the major complication after abdominal surgery. Peritoneal adhesion is an abnormal connective tissue that occurs between two tissues that have been damaged during the surgery [1, 2]. The peritoneum gets harmed and forms a temporary matrix during the surgery. After several hours, this provisional matrix becomes a clot, which can be destroyed by various factors such as macrophages and fibrinolysin enzymes.

Following the clot formation after 72 hours, the fibroblasts of the underlying tissues migrate into the clot and provide a field for forming sticky tissue [3, 4]. It has been emphasised that inflammation, free radicals, hypoxia, coagulation, and fibrinolysis are the main pathophysiological reasons responsible for forming peritoneal adhesion [2, 5].

Plasminogen activator (PA) is a protease that converts plasminogen into plasmin and prevents mesothelial cell adhesion [3]. Numerous inflammatory cytokines are released following the peritoneal injury, such as interferon-gamma (IFN-*γ*), interleukin-4 (IL-4), IL-10, IL-6, tumour necrosis factor-alpha (TNF-*α*), and prostaglandin E2 (PGE-2) [6–9]. These inflammatory cytokines play an essential role in the development of peritoneal adhesion. Transforming growth factor-beta (TGF-*β*) is expressed by adhesion fibroblasts and mesothelial cells, which lead to adhesion formation and fibrosis [7, 10]. Additionally, vascular endothelial growth factor (VEGF) is another important marker in angiogenesis, wound healing, and adhesion formation [2, 11].

The surgical technique is the first method for adhesion treatment; however, it is insufficient alone [12]. Other therapeutic approaches have been studied, such as barrier therapy [13, 14] and gene therapy [15]. However, there is still no approved method for the treatment or prevention of adhesion, although a high prevalence of postoperative adhesions.

*Crocus sativus* (*C. sativus*), popularly named *C. sativus*, is a small plant belonging to Iridaceae. Crocin, crocetin, and safranal are the major ingredients of *C. sativus* [16]. Several pharmacological properties of *C. sativus* have been reported, including the antioxidant [17, 18], anticancer, anti-inflammatory [19, 20], anti-ischemia, and cardioprotective [21, 22] effects.

To our knowledge, there is no study evaluating the protective effects of *C. sativus* extract on preventing postoperative intra-abdominal adhesions. Therefore, in the present study, we aimed to determine the anti-inflammatory and antioxidant effects of *Crocus sativus* on the formation and prevention of postoperative abdominal adhesions in a rat model of peritoneal adhesion.

## 2. Material and Methods

### 2.1. Drugs and Chemicals

Ethanol, methanol, acetonitrile, formic acid, dimethyl sulfoxide, ammonium chloride, HCl, and dexamethasone were purchased from Sigma®, USA. Ketamine and xylazine were obtained from ChemiDarou®, Iran. The injectable normal saline serum was also prepared from Samen®, Iran. Furthermore, enzyme-linked immunosorbent assay (ELISA) kits of IL-4, IL-10, IL-6, *TN*F-*α*, PGE_2_, TGF-*β*, tissue plasminogen activator (tPA), and plasminogen activator inhibitor (PAI) and VEGF were purchased from Bender Med®, Germany. Dulbecco's modified Eagle's medium/F12 (DMEM/F12) culture media, fetal bovine serum (FBS), penicillin plus streptomycin (pen/strep), dimethyl sulfoxide (DMSO), and other chemicals used were of cell culture and analytical grade from Sigma-Aldrich (St. Louis, MO, USA). Recombinant mouse TGF-*β*1 (5231LC) was obtained from Cell Signaling Technology, Inc. The levels of malondialdehyde (MDA) as an oxidative marker and glutathione (GSH) as an antioxidative marker were measured using commercially available biochemistry kits (ZellBio®, Germany).

### 2.2. Preparation of C*. sativus* Extract

C. *sativus* was prepared from *C. sativus* farms of Qaen (33°43′33.02^″^N 59°11′21.65^″^E, South Khorasan, Iran) and identified by the herbarium of Ferdowsi University of Mashhad (herbarium No. 293-0303-1). The 70% *v*/*v* hydroethanolic extract of C. *sativus* was prepared using the maceration method as described previously [23]. Briefly, 10 g of its ground petal stigma was incubated with 400 ml of 70% *v*/*v* ethanol in a macerated extractor for 72 h. The prepared extract was dried in a rotatory evaporator and stored at -20°C until use.

### 2.3. High-Performance Liquid Chromatography- (HPLC-) Mass Spectrometry (MS) Apparatus and the Extracted Analysis

The LC-MS analysis was performed in an AB SCIEX QTRAP (Shimadzu) liquid chromatography coupled with a triple quadrupole Mass Spectrometer. Liquid chromatography separation was performed on a Supelco C18 (15 mm × 2.1 mm × 3 *μ*m) column. MS analysis was carried out in both negative and positive modes of ionisation to monitor as many ions as possible and to ensure that the most significant number of metabolites extracted from the *C. sativus* sample was detected. The analysis was done at a flow rate of 0.2 ml/min. The gradient analysis started with 100% of 0.4% aqueous formic acid, isocratic conditions were maintained for 1 min, and then a 14 min linear gradient to 40% acetonitrile with 0.4% formic acid was applied. From 14 to 35 min, the acidified acetonitrile was increased to 100%, followed by 5 min of 100% acidified acetonitrile and 5 min at the start conditions to reequilibrate the column. The mass spectra were acquired in a range of 100 to 1500 within the 45 minutes scan time. Mass feature extraction of the acquired LC-MS data and maximum detection of peaks was done using the MZmine analysis software package, version 2.3.

### 2.4. *In Vivo* Study

#### 2.4.1. Animals

Seventy male Wistar-Albino rats weighing 250 ± 15 g (six weeks old) were purchased from the animal laboratory unit of Faculty of Medicine, Mashhad University of Medical Sciences, Mashhad, Iran. Rats were housed in separated standard cages and ventilated room with a 12/12 h natural light-dark cycle, 60 ± 3% humidity, and temperature of 21 ± 2°C. They had free access to food and taped water before and during the experiments. More appropriate hygiene was provided with continuous cleaning and removal of faeces and spilt feeds from cages daily. All animals received human care in compliance with institutional guidelines.

#### 2.4.2. Surgical Procedure

The ethical committee approved all animal-related procedures based on the guidelines of animal experiments in Mashhad University of Medical Sciences (ethical approved code http://IR.MUMS.fm.REC.1395.950309, Approval Date: 2017-03-01). The surgical method was accomplished as previously described [2, 24, 25]. In summary, animals received 100 mg/kg of ketamine and 10 mg/kg of xylazine intraperitoneally (i.p.) for anaesthesia. Following the skin's shaving and disinfection with alcohol and iodine solution, a three-centimeter incision was carefully done to reach the abdominal cavity. For intra-abdominal adhesion induction in rats, the peritoneal abrasion method was performed as one side of the middle abdominal incision was gently abraded using a soft sterilised paper polisher until the cecum provided an opaque presentation with fine petechiae. Afterwards, the peritoneum and the injured area were washed by 2 ml of the extract or vehicle. After the intervention, the cecum was returned to the abdomen and abdomen wall then closed with 4-0 poly-gelatine suture. The procedure lasted to a maximum of 10 minutes. After surgery, rats were kept in their cages in the recovery room for seven days. All treatments were done by lavage in the abraded and whole surgical zone with a 2 ml syringe. Furthermore, all rats received a single dose of antibiotic cefazolin (300 mg/kg intramuscularly; i.m.) immediately after ending the surgery to prevent possible wound infection [26–28].

#### 2.4.3. Experimental Groups

Seventy male Wistar rats were randomly divided into seven groups containing ten animals and grouped as follows:

1. Group 1: normal—rats received neither surgical nor intervention procedures.
2. Group 2: control—rats received surgical and peritoneal adhesion procedures without treatment.
3. Group 3: vehicle—rats received surgical and peritoneal adhesion procedures and were treated with 2 ml of the vehicle (the vehicle was sterilised distilled water containing 5% v/v of tween 80 [2]).
4. Group 4: pirfenidone (PFD)—rats received surgical and peritoneal adhesion procedures and were treated with 2 ml of the 7.5% *w*/*v* of PFD (approximately 600 mg/kg or 150 mg/animal [29–31]), as positive control and the antifibrotic agent [29–31].
5. Groups 5, 6, and 7: C. sativus extracts (S)—rats received surgical and peritoneal adhesion procedures and were treated with 2 ml of either 0.125% *w*/*v*, 0. 25% *w*/*v*, or 0.5% *w*/*v* of the extract (approximately 12.5, 25, and 50 mg/kg, respectively); the concentrations were chosen based on our preliminarily experiment.

#### 2.4.4. Assessment of the Macroscopic Adhesion Grade

On the seventh day after the surgery, rats underwent a second laparotomy. Thereafter, two independent researchers blind to the protocol assessed the adhesion grading using the score published by Nair et al. [32] (Table 1). Additionally, cecum and peritoneal lavage fluid were collected for the measurement of inflammatory, fibrotic, and oxidative biomarkers.

#### 2.4.5. Histological Assessment

In the current experiment, paraffin-embedded histological sections were stained by Masson's trichrome staining to assess the extent and distribution of fibrosis in rats' peritoneal tissue as described in previous studies [33–35]. In this regard, after removing formalin and washing with distilled water three times, the tissues were transferred to different alcohol concentrations (50-100%) for some minutes. Tissue sections were observed with magnifications of 4x, 20x, and 40x using a Nikon E-1000 microscope (Japan) under bright-field optics.

#### 2.4.6. Evaluation of Oxidative Parameters

The levels of MDA, as an oxidative marker, and GSH, as an antioxidative marker, were measured in the peritoneal fluid using biochemistry kits (ZellBio®, Germany) according to the manufacturer's manuals [36, 37].

#### 2.4.7. Assessment of Inflammatory and Anti-Inflammatory Biomarkers

The levels of TNF-*α*, IL-6, and PGE_2_, as inflammatory markers, and IL-4 and IL-10, as anti-inflammatory markers, were evaluated in peritoneal lavage fluid by ELISA kits (Bender Med®, Germany) according to the manufacturer's instruction [38, 39].

#### 2.4.8. Evaluation of Fibrosis and Angiogenesis Biomarkers and Tissue Plasminogen Activator (tPA) and Plasminogen Activator Inhibitor (PAI)

According to the manufacturer's instruction, the concentrations of fibrosis biomarkers (TGF-*β*) and angiogenesis marker (VEGF) of peritoneal fluid specimens were assessed by the relevant ELISA kits. Additionally, according to the manufacturer's instruction, the levels of tPA, which digests fibrin substrates, and PAI were also evaluated in peritoneal lavage fluid by ELISA kits. Subsequently, the tPA/PAI ratio was calculated by dividing the level of tPA by PAI level. The levels of cytokines were reported as pg/mg protein.

### 2.5. *In Vitro* Study

#### 2.5.1. Cell Culture Condition

Murine macrophage cell line, RAW 264.7, was purchased from Pasture Institute, Tehran, Iran. The cells were cultured in DMEM/F12 enriched with 10% *v*/*v* foetal bovine serum (FBS), 100 IU/ml penicillin, and 100 *μ*g/ml streptomycin at 37°C in a humidified atmosphere with 5% *v*/*v* CO_2_ [40].

#### 2.5.2. Proliferation Assay

To investigate that *C. sativus* extract had no cytotoxicity and inhibitory effects on RAW 264.7 cells, the cells were cultured at a density of 3 × 10^3^ cells/well in 96-flat well plates and incubated overnight [40]. Thereafter, the cells were incubated with different concentrations of *C. sativus* extract (100, 200, and 400 *μ*g/ml, according to the preliminary evaluation), PFD (100 *μ*g/ml, as a positive control group, [41]), or vehicle (contained 0.1% dimethyl sulfoxide, DMSO) for 48 h, at 37°C and 5% *v*/*v* CO_2_.

In another set of experiments, we assessed the effects of different concentrations of *C. sativus* extract (100, 200, and 400 *μ*g/ml, according to the preliminary evaluation), PFD (100 *μ*g/ml, as a positive control group, [41]), or vehicle (contained 0.1% dimethyl sulfoxide, DMSO) in the presence of recombinant mouse TGF-*β*1 stimulation (20 ng/ml [42]) on cell proliferation. In this regard, the cells (3 × 10^3^) were incubated with the extract, PFD, vehicle, or medium for 24 h and then coincubated with TGF-*β*1 (20 ng/ml [42]) for another 24 h, at 37°C and 5% *v*/*v* CO_2_. Afterwards, cell proliferation was also assessed by the MTT method.

Finally, cell proliferation was assessed using the 3-(4,5-dimethylthiazol-2-yl)-2,5-diphenyl-2H-tetrazolium bromide (MTT) method. Briefly, 10 *μ*l of MTT solution with a final concentration of 5 mg/ml was appended to each well to be incubated for 3 h. After discarding the medium culture (DMEM/F12), 100 *μ*l of DMSO was used to dissolve the formed formazan crystals. The absorption of the 96-flat wells plate was recorded by ELISA reader (Awareness Inc., USA) at 570 nm and 620 nm [40, 43].

#### 2.5.3. Assessment of Secretory Cytokines Levels and Intracellular Levels of iNOS and Arg-1

According to the manufacturer's instructions, the anti-inflammatory (IL-10) levels and inflammatory cytokine (TNF-*α*) and angiogenesis factor (VEGF) were measured by the ELISA-based method. The cells were cultured in 6-well plates (2 × 10^6^ cells/each well) and incubated with different concentrations of *C. sativus* extract (100, 200, and 400 *μ*g/ml, according to the preliminary evaluation), PFD (100 *μ*g/ml, as a positive control group, [41]), or vehicle (contained 0.1% dimethyl sulfoxide, DMSO) in the presence of recombinant mouse TGF-*β*1 stimulation (20 ng/ml, providing M2 phenotype cells [42]) for 24 h and then coincubated with TGF-*β*1 (20 ng/ml [42]) for another 24 h, at 37°C in a 5% *v*/*v* CO_2_ incubator. Finally, the supernatants were collected to measure the levels of cytokines. The levels of cytokines were reported as pg/mg protein. Moreover, the cells were collected and lysed using a lysis buffer and then homogenised (DIAX 100, Heidolph, Schwabach, Germany) on the cold water (0-4°C) for 2–3 min along with vortexing (every 30 sec). The samples were centrifuged at 12,000 g for 10 min at 4°C, and 50 *μ*l of supernatants had then undergone an assessment. The levels of iNOS and Arg-1 were reported as ng/mg protein.

### 2.6. Statistical Analysis

Data were analysed using GraphPad Prism (version 6.01) software and presented according to the nature of parametric or nonparametric as the means ± SEM or median ± interquartile range, respectively. *P* values ≤ 0.001, 0.01, and 0.05 were statistically considered significant. For parametric data, one-way ANOVA was performed with the following Tukey's Kramer *post hoc* test. However, for nonparametric data (adhesion score), the Kruskal-Wallis test was done following Dunn's multiple comparisons posttest. The data and statistical analysis comply with the recommendations on experimental design, analysis [44], and data sharing and preclinical pharmacology presentation [45, 46].

## 3. Results

### 3.1. LC-MS Analysis and Characterisation of *C. sativus* L. Extract

**Figure 1:**
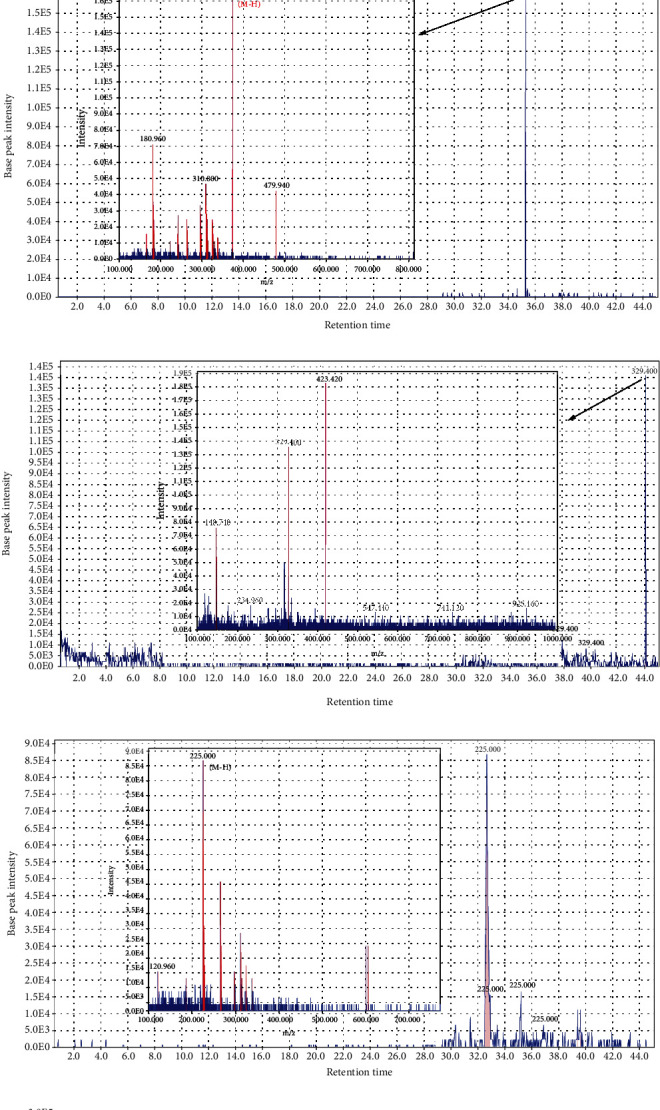
(a) The total ion chromatogram of *C. sativus* (*Crocus sativus* L.) using LC-MS in the positive mode. (b) The total ion chromatogram of *C. sativus* (*Crocus sativus* L.) using LC-MS in the negative mode. (c) Chromatogram of 4-(*α*-D-glucopyranosyl)-2, 6,6-trimethyl-1-cyclohexene-1-carboxaldehyde (picrocrocin), and corresponding mass adduct, [M-H], at *m*/*z* 375.2. (d) Chromatogram of dihydrojasmonic acid, methyl ester, and corresponding mass adduct, [M-H], at *m*/*z* 225.0. (e) Chromatogram of dihydrojasmonic acid, methyl ester and corresponding mass adduct, [M-H], at *m*/*z* 225.0. (f) Chromatogram of naringenin and corresponding mass adduct, [M+H], at *m*/*z* 273.3. (g) Chromatogram of Kaempferol 3-sophoroside-7-glucoside and corresponding mass adduct, [M+H], at *m*/*z* 772.2.

Collectively, 35 compounds (in ESI+ and ESI−) were identified in the hydroethanolic extract of *C. sativus* L., including flavonoids and crocins (crocin and its derivatives). Data concerning the identification of the compounds are shown in Tables 2 and 3. The total ion chromatograms of *C. sativus* L. extract in both ESI+ and ESI− modes are shown in Figures 1(a) and 1(b), respectively. The MS spectral data were compared with the reported compounds in some previous literature. Figures 1(a)–1(f) are examples of extracted ion chromatograms from the total ion chromatogram and its related mass. Some flavonoids, including quercetin 3-orutinosylrhamnoside, quercetin 3-O-rutinoside, Kaempferol 3-glucoside, tamarixetin 3-O-bihexoside, rhamnetin, and naringenin, were detected in *C. sativus* L. extract. Apocarotenoids, including crocin, crocetin, and their derivatives, apart from imparting colours to *C. sativus*, also have antioxidant properties (40).

### 3.2. *In Vivo* Results

#### 3.2.1. The Effect of *C. sativus* and PFD on Adhesion Score

**Figure 2:**
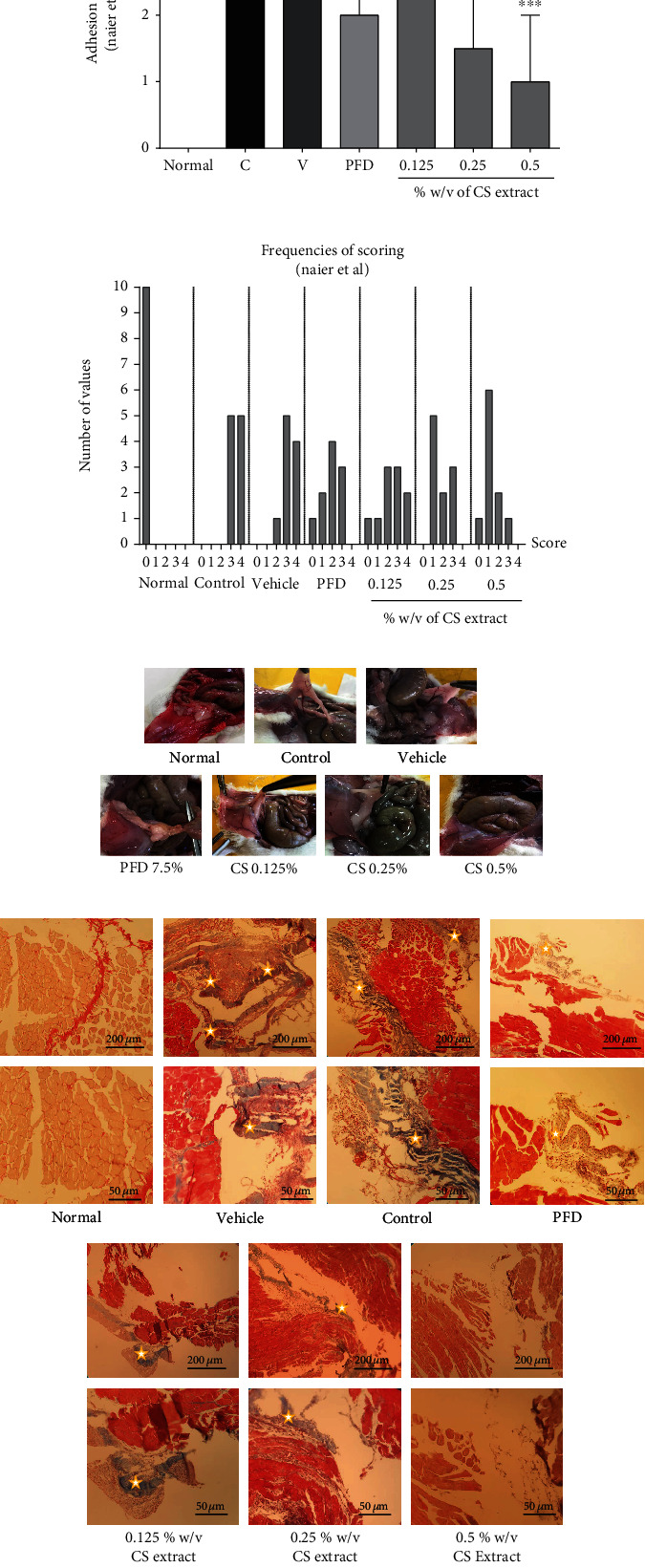
The effects of different concentrations of *C. sativus* (0.125, 0.25, and 0.5% *w*/*v*) and PFD (7.5% *w*/*v*) on adhesion score (a) and frequencies of scoring (b) following postoperational-induced peritoneal adhesion. (c) The images of adhesion bands in different groups. (d) The effects of different doses of *C. sativus* extract on adhesion formation and collagen deposition by histopathological evaluation using Masson's trichrome staining; blue colour intensities (marked with white stars) represent fibrosis and collagen deposition. Data were presented as the median ± interquartile range (IQR) (*n* = 10). ^∗∗∗^*P* < 0.001 and ^∗∗^*P* < 0.01 compared to the control group.

The adhesion scores in both the control and vehicle groups were increased compared to those in the normal group (*P* < 0.001 for both cases, Figures 2(a)–2(c)). Treatment with PFD (7.5% *w*/*v*, *P* < 0.01) and *C. sativus* (0.25% *w*/*v*, *P* < 0.01, and 0.5% *w*/*v*, *P* < 0.001) significantly attenuated the levels of adhesion score compared to the control group (Figure 2(a)). The frequencies of adhesion score are indicated in Figure 2(b) according to the Nair et al. scoring system.  Figure 2(c) shows the samples of the adhesion band in each group.

#### 3.2.2. The Effects of PO Extract on Histopathological Alteration of Peritoneal Fibrosis

Our histopathological results showed the levels of tissue fibrosis and collagen deposition (blue colour) in both the vehicle and control groups (Figure 2(d)). On the contrary, the blue colour's intensities were notably lower in all doses of the extract groups and PFD as a positive control than the control group (Figure 2(d)).

#### 3.2.3. The Effect of *C. sativus* and PFD on Anti-Inflammatory Biomarkers

**Figure 3:**
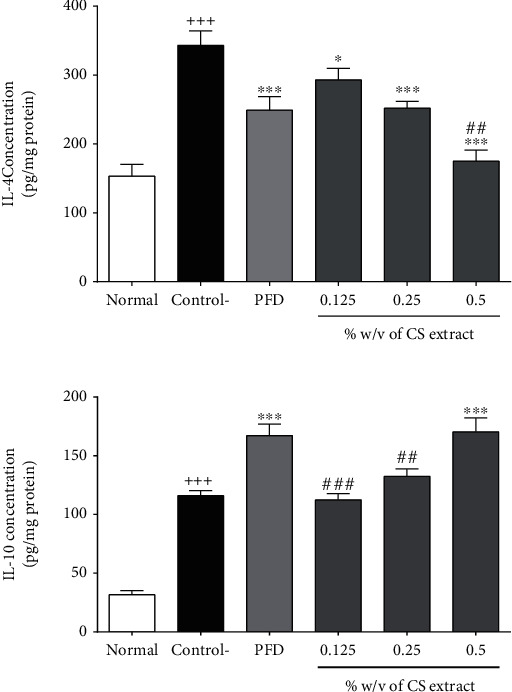
The effect of different concentrations of *C. sativus* (0.125, 0.25, and 0.5% *w*/*v*) and PFD (7.5% *w*/*v*) on IL-4 (a) and IL-10 (b) levels following the postoperational-induced peritoneal adhesion. Data were presented as the mean ± SEM (*n* = 8). ^+++^*P* < 0.001 compared to the normal group, ^∗∗∗^*P* < 0.001 compared to the control group, and ^###^*P* < 0.001 and ^##^*P* < 0.01 compared to the PFD group. The lines represent a significant difference between the three *C. sativus* groups.

Following the peritoneal adhesion induction, the levels of IL-4 and IL-10 were markedly increased in the control group compared to the normal group (*P* < 0.001 for both cases, Figures 3(a) and 3(b)). The level of IL-4 was notably diminished by treatment with either all concentrations of *C. sativus* (0.125% *w*/*v*, *P* < 0.05, 0.25% *w*/*v*, *P* < 0.001, and 0.5% *w*/*v*, *P* < 0.001, Figure 3(a)) or PFD (7.5% *w*/*v*, *P* < 0.001, Figure 3(a)) compared to the control group. The extract of *C. sativus* (0.5% *w*/*v*, *P* < 0.01, Figure 3(a)) significantly reduced IL-4 level in peritoneal lavage fluid than that in the PFD-treated group (7.5% *w*/*v*). Both PFD (7.5% *w*/*v*, *P* < 0.001, Figure 3(b)) and *C. sativus* (0.5% *w*/*v*, *P* < 0.001, Figure 3(b)) considerably increased the level of IL-10 in peritoneal lavage fluid.

#### 3.2.4. The Effect of *C. sativus* and PFD on the Levels of tPA, PAI, and tPA/PAI Ratio

**Figure 4:**
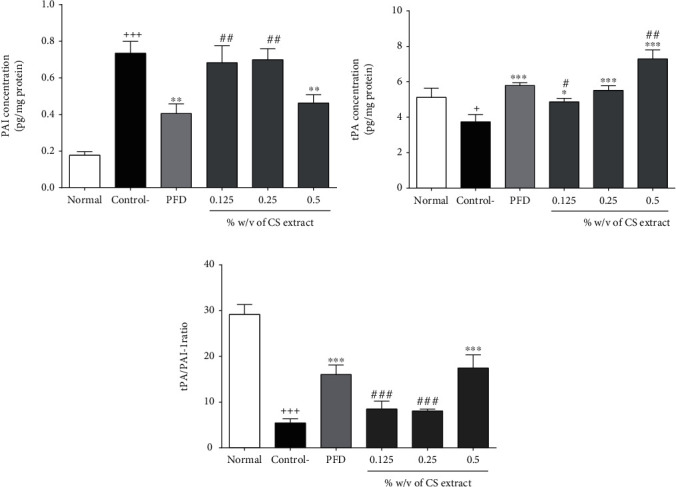
The effect of different concentrations of *C. sativus* (0.125, 0.25, and 0.5% *w*/*v*) and PFD (7.5% *w*/*v*) on PIA (a), tPA (b), and tPA/PAI ratio (c) levels following post-operational-induced peritoneal adhesion. Data were presented as the mean ± SEM (*n* = 8). ^+++^*P* < 0.001 and *P* < 0.01 compared to the normal group, ^∗∗∗^*P* < 0.001 to^∗^*P* < 0.05 compared to the control group, and ^###^*P* < 0.001 to ^#^*P* < 0.05 compared to the PFD group. The lines represent a significant difference between the three *C. sativus* groups.

The levels of tPA (*P* < 0.05, Figure 4(b)) and tPA/PAI ratio (*P* < 0.001, Figure 4(c)) were diminished, but PAI level (*P* < 0.001, Figure 4(a)) was increased in the control group compared to the normal group. Treatment with *C. sativus* (0.125%*w*/*v*, *P* < 0.05, 0.5%*w*/*v*, *P* < 0.001, and 0.25%*w*/*v*, *P* < 0.001) and PFD (7.5%*w*/*v*, *P* < 0.001) significantly increased the tPA level in a concentration-dependent manner (Figure 4(b)). Treatment with a high concentration of *C. sativus* (0.5% *w*/*v*) and PFD (7.5% *w*/*v*) markedly decreased PAI level (*P* < 0.01 for both cases, Figure 4(a)) and significantly increased tPA/PAI ratio (*P* < 0.001 for both cases, Figure 4(c)) in the peritoneal lavage fluid compared to the control group.

#### 3.2.5. The Effect of *C. sativus* and PFD on Fibrotic (TGF-*β*1) and Angiogenesis (VEGF) Parameters

**Figure 5:**
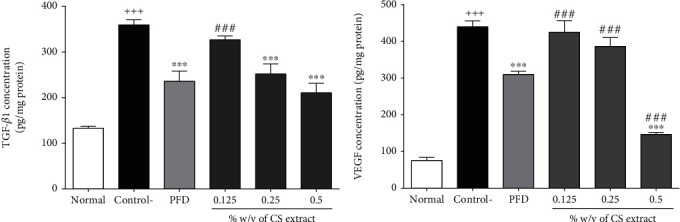
The effect of different concentrations of *C. sativus* (0.125, 0.25, and 0.5% *w*/*v*) and PFD (7.5% *w*/*v*) on TGF-*β*1 (a) and VEGF (b) levels following postoperational-induced peritoneal adhesion. Data were presented as the mean ± SEM (*n* = 8). ^+++^*P* < 0.001 compared to the normal group, ^∗∗∗^*P* < 0.001 compared to the control group, and ^###^*P* < 0.001 compared to the PFD group. The lines represent a significant difference between the three *C. sativus* groups.

The levels of TGF-*β*1 (*P* < 0.001, Figure 5(a)) and VEGF (*P* < 0.001, Figure 5(a)) were significantly increased in the control group compared to the normal group. Two higher concentrations of *C. sativus* (0.25% *w*/*v*, *P* < 0.001, and 0.5% *w*/*v*, *P* < 0.001) and PFD (7.5% *w*/*v*, *P* < 0.001) significantly reduced the concentration of TGF-*β*1 compared to the control group (Figure 5(a)). However, the level of VEGF was significantly decreased by administration of either *C. sativus* (0.5% *w*/*v*, *P* < 0.001) or PFD (7.5% *w*/*v*, *P* < 0.001), compared to the control group (Figure 5(b)).

#### 3.2.6. The Effect of *C. sativus* and PFD on Inflammatory Parameters (TNF-*α*, IL-6, and PGE_2_)

**Figure 6:**
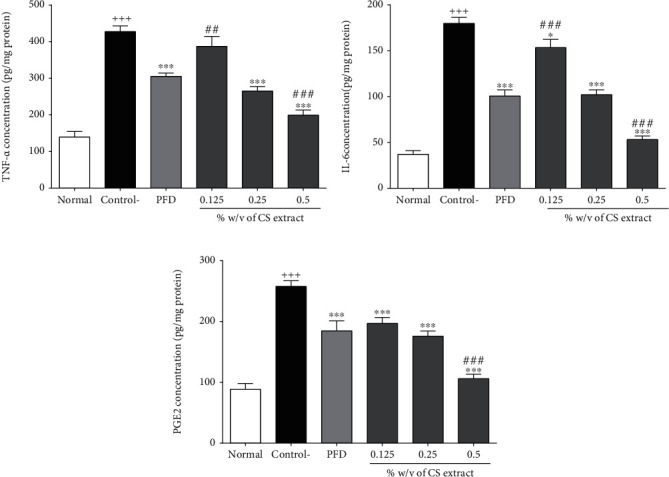
The effect of different concentrations of *C. sativus* (0.125, 0.25, and 0.5% *w*/*v*) and PFD (7.5% *w*/*v*) on TNF-*α* (a), IL-6 (b), and PGE_2_ (c) levels following postoperational-induced peritoneal adhesion. Data were presented as the mean ± SEM (*n* = 8). ^+++^*P* < 0.001 compared to the normal group, ^∗∗∗^*P* < 0.001 and^∗^*P* < 0.05 compared to the control group, and ^###^*P* < 0.001 and ^##^*P* < 0.01 compared to the PFD group.

All inflammatory parameters (TNF-*α*, IL-6, and PGE_2_) were increased in the control group compared to the normal group (*P* < 0.001 for all cases, Figures 6(a)–6(c)). All three concentrations of *C. sativus* (0.125, 0.25, and 0.5% *w*/*v*) and PFD (7.5% *w*/*v*) decreased IL-6 (*P* < 0.001-0.05 for all cases, Figure 6(b)) and PGE_2_ (*P* < 0.001 for all cases, Figure 6(c)) levels. Moreover, *C. sativus* (0.25, 0.5% *w*/*v*) and PFD were diminished TNF-*α* concentration compared to the control group in peritoneal lavage fluid (*P* < 0.001 for all cases, Figure 6(c)).

#### 3.2.7. The Effect of *C. sativus* and PFD on MDA and GSH

**Figure 7:**
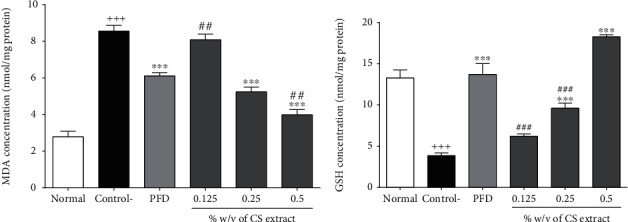
The effect of different concentrations of *C. sativus* (0.125, 0.25, and 0.5% *w*/*v*) and PFD (7.5% *w*/*v*) on MDA (a) and GSH (b) levels following postoperational-induced peritoneal adhesion. Data were presented as the mean ± SEM (*n* = 8). ^+++^*P* < 0.001 and ^+^*P* < 0.05 compared to the normal group, ^∗∗∗^*P* < 0.001 compared to the control group, and ^###^*P* < 0.001 and ^##^*P* < 0.01 compared to the PFD group. The lines represent a significant difference between the three *C. sativus* groups. The lines represent a significant difference between the two groups shown.

The concentrations of MDA (*P* < 0.001, Figure 7(a)) and GSH (*P* < 0.001, Figure 7(b)) were significantly increased and decreased in the control group compared to the normal group, respectively. The levels of MDA and GSH, respectively, diminished and increased following treatment with *C. sativus* (0.25, 0.5%*w*/*v*) and PFD (7.5%*w*/*v*) in comparison to the control group in peritoneal lavage fluid (*P* < 0.001 for all cases, Figures 7(a) and 7(b)).

### 3.3. *In Vitro* Results

#### 3.3.1. The Effect of *C. sativus* Extract and PFD on Cell Proliferation

**Figure 8:**
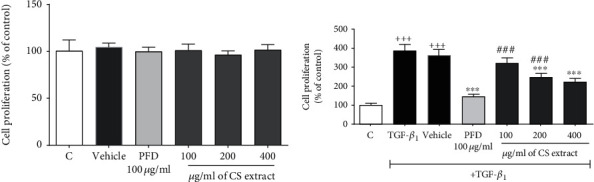
The effects of *C. sativus* extract (100, 200, and 400 *μ*g/ml) and PFD (100 *μ*g/ml) on proliferation level of RAW 264.7 macrophage cells. Cell proliferation without stimulation (a) and cell proliferation with TGF-*β* stimulation (20 ng/ml) (b). Data were presented as the mean ± SEM (*n* = 6). ^+++^*P* < 0.001, compared with the control group; ^∗∗∗^*P* < 0.001, compared with the TGF-*β*_1_ group; ^###^*P* < 0.001, compared with PFD group. The lines represent a significant difference between the two groups shown.

In the absence of TGF-*β*_1_ stimulation, no significant changes were found in cell proliferation between the groups treated with vehicle, *C. sativus* extract (100, 200, and 400 *μ*g/ml) and PFD (100 *μ*g/ml) and the control group (Figure 8(a)). In the presence of TGF-*β*_1_ stimulation (20 ng/ml), the levels of cell proliferation were significantly increased in both vehicle-treated and TGF-*β*_1_ groups compared to the respected control group (*P* < 0.001 for both cases, Figure 8(b)). Pretreatment with *C. sativus* extract (200 and 400 *μ*g/ml) and PFD (100 *μ*g/ml) significantly decreased the level of cell proliferation compared to the TGF-*β*1-treated alone group (*P* < 0.001 for all cases, Figure 8(b)). The potential protective effects of *C. sativus* extract (100 and 200 *μ*g/ml) were lower than those of PFD (100 *μ*g/ml) on decreasing the TGF-*β*_1_-induced cell hyperproliferation (*P* < 0.001 for both case, Figure 8(b)).

#### 3.3.2. The Effect of *C. sativus* Extract and PFD on TNF-*α*, IL-10, and VEGF

**Figure 9:**
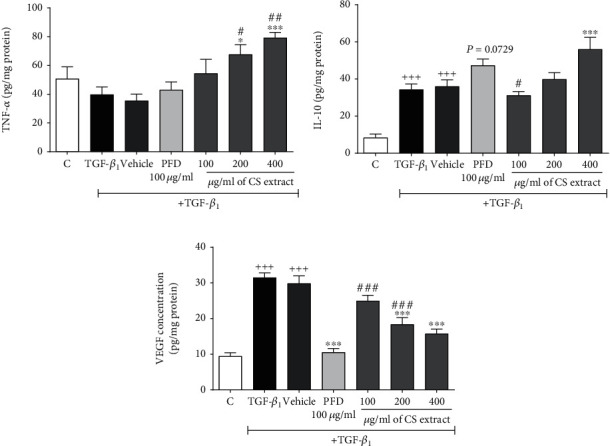
Effects of *C. sativus* extract (100, 200, and 400 *μ*g/ml) and PFD (100 *μ*g/ml) on the mRNA expression levels of proinflammatory factors of RAW 264.7 macrophage cells, including TNF-*α* (a), IL-10 (b), and VEGF (c) in the presence of TGF-*β* stimulation (20 ng/ml). Data were presented as the mean ± SEM (*n* = 6). ^+++^*P* < 0.001, compared with the control group; ^∗∗∗^*P* < 0.001 and ^∗^*P* < 0.05 compared with the TGF-*β*_1_ group; ^###^*P* < 0.001 to ^#^*P* < 0.05 compared with the PFD group. PFD was increased TNF-*α* level, but it had no significant difference compared to the TGF-*β*_1_ group (*P* = 0.0729).

In the presence of TGF-*β*_1_ stimulation (20 ng/ml), the TNF-*α* level had no considerable changes in both the TGF-*β*1 and vehicle groups compared to the control group (Figure 9(a)). In contrast, IL-10 (*P* < 0.001 for both cases, Figure 9(b)) and VEGF (*P* < 0.001 for both cases, Figure 9(c)) levels were significantly increased in TGF-*β*1 and vehicle groups compared to the control group. Premedication with PFD (100 *μ*g/ml) enhanced the TNF-*α* level, but it had no statistically significant difference compared to the TGF-*β*1 group (*P* = 0.0729, Figure 9(b)). Pretreatment with *C. sativus* extract (200 and 400 *μ*g/ml) significantly increased TNF-*α* (*P* < 0.05 and *P* < 0.001, respectively, Figure 9(a)) level and notably decreased VGEF (*P* < 0.001 for both cases, Figure 9(c)) level compared to the TGF-*β*1 group. However, pretreatment with a high concentration of *C. sativus* extract (400 *μ*g/ml) significantly increased IL-10 level compared to the TGF-*β*1 group (*P* < 0.001, Figure 9(c)).

#### 3.3.3. The Effect of *C. sativus* Extract and PFD on Protein Levels of iNOS and Arg-1 and iNOS/Arg-1 Ratio

**Figure 10:**
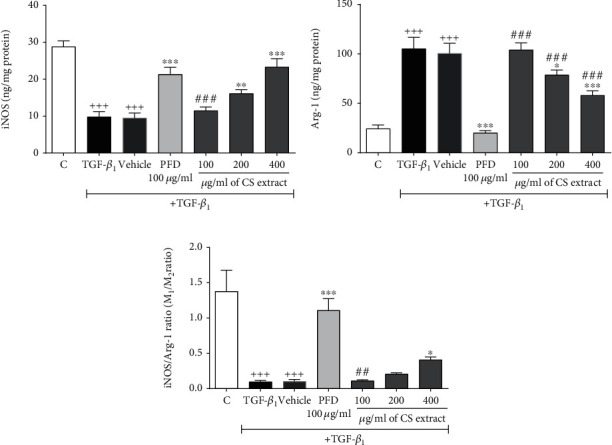
Effects of *C. sativus* extract (100, 200, and 400 *μ*g/ml) and PFD (100 *μ*g/ml) on the iNOS and Arg-1 levels iNOS/Arg-1 ratio of RAW 264.7 macrophage cells. (a) iNOS, (b) Arg-1, and (c) iNOS/Arg-1 ratio in the presence of TGF-*β* stimulation (20 ng/ml). Data were presented as the mean ± SEM (*n* = 6). ^+++^*P* < 0.001, compared with the control group; ^∗∗∗^*P* < 0.001 and ^∗^*P* < 0.05 compared with the TGF-*β*_1_ group; ^###^*P* < 0.001 and ^##^*P* < 0.01 compared with the PFD group.

In the presence of TGF-*β*_1_ stimulation (20 ng/ml), iNOS level (*P* < 0.001, Figure 10(a)) and iNOS/Arg-1 ratio (*P* < 0.001, Figure 10(c)) were significantly diminished, but Arg-1 level (*P* < 0.001, Figure 10(b)) was meaningfully increased in the TGF-*β*1 and vehicle groups compared to the control group. Pretreatment with *C. sativus* extract (200 *μ*g/ml, *P* < 0.01, and 400 *μ*g/ml, *P* < 0.001) and PFD (100 *μ*g/ml, *P* < 0.001) significantly increased iNOS level (Figure 10(a)) compared to the TGF-*β*1 group. On the contrary, *C. sativus* extract (200 *μ*g/ml, *P* < 0.05, and 400 *μ*g/ml, *P* < 0.001) and PFD (100 *μ*g/ml, *P* < 0.001) significantly reduced Arg-1 level (Figure 10(b)) in comparison to the TGF-*β*1 group. Our results indicated that the highest concentration of *C. sativus* extract (400 *μ*g/ml, *P* < 0.05) and PFD (100 *μ*g/ml, *P* < 0.001) could increase the iNOS/Arg-1 ratio compared to the TGF-*β*1 group (Figure 10(c)).

## 4. Discussion

The present study evaluated the protective effects of hydroethanolic extract of *C. sativus* stigma against postoperational-induced peritoneal adhesion in a rat model. As a result, the current study demonstrated that *C. sativus* extract ameliorates postoperational-induced peritoneal adhesion development through attenuating oxidative stress (MDA), inflammatory mediators (IL-6, TNF-*α*, and PGE_2_), and fibrosis (TGF-*β*1, IL-4, and PAI) and angiogenesis (VEGF) markers, while propagating antioxidant (GSH), anti-inflammatory (IL-10), and fibrinolytic (tPA) markers and tPA/PAI ratio. Moreover, we assessed the protective and antifibrotic effects of the extract against TGF-*β*1-induced fibrosis in RAW 264.7 murine macrophage cell line. Briefly, we revealed that the extract, without any toxicity, modulated the levels of cell proliferation and inflammatory (TNF-*α*), angiogenesis (VEGF), anti-inflammatory (IL-10), M1 (iNOS), and M2 (Arg-1) biomarkers and iNOS/Arg-1 ratio towards antifibrotic M1 phenotype of macrophage, in a concentration-dependent manner.

Numerous models have been suggested to evaluate postoperative peritoneal adhesion, including uterine horn damage, bacterial infection, and scarping model [47, 48]. In the current study, we used the scraping model due to the most similarity between the adhesion development by this model and abdominopelvic surgery [49, 50]. Furthermore, we scored the adhesions from zero to four using the Nair et al. and adhesion scheme scoring methods [25, 32, 50]. Our macroscopic data revealed that the adhesion score was significantly increased in the control group, while *C. sativus* (0.25 and 0.5% *w*/*v*) concentration-dependently reduced the adhesion formation following postoperational-induced peritoneal adhesion in the rat. Our previous study also reported that the adhesion score is enhanced in the control group that received postoperative-induced peritoneal adhesion and decreased following the interventions, such as propolis, honey, and *Rosmarinus officinalis* treatments [2, 24, 25, 32].

In the present study, we used pirfenidone (PFD), a well-known antifibrotic medicine, as a positive control. We showed that PFD (7.5% *w*/*v*) provided a significant decrement in adhesion score, MDA, TNF-*α*, PGE_2_, IL-6, IL-4, TGF-*β*, VEGF, and PAI levels, while making a significant increment in GSH, IL-10, and tPA levels as well as tPA/PAI ratio following postoperational-induced adhesion in the rat. Moreover, following the TGF-*β*1 stimulation, our cellular results also revealed that PFD (100 *μ*g/ml) significantly reduced the levels of cell proliferation, VEGF, and Arg-1 but notably enhanced IL-10, iNOS, and iNOS/Arg-1 ratio (M1/M2 marker) and polarized the macrophage from fibrotic phenotype towards antifibrotic M1 cells. Following our results, Bayhan et al. indicated that oral administration of PFD (500 mg/kg po~6.25% *w*/*v*) for two weeks significantly reduced adhesions grade and the protein concentrations and mRNA expression levels of matrix metallopeptidase-9 (MMP-9), tissue inhibitor of metalloproteinase-1 (TIMP-1), tumour necrosis factor-alpha (TNF-*α*), and TGF-*β*1 [29]. Similarly, Ozbilgin and coworkers reported the protective effects of PFD (150 mg/animal~2 ml of 7.5% *w*/*v*) against peritoneal adhesion. In fact, they showed that PFD as the same concentration which used in our study (2 ml of 7.5% *w*/*v*) significantly diminished the peritoneal adhesion by decreasing the Th2 lymphocytes as fibrotic cells and increasing the Th1 lymphocytes as antifibrotic cells [31]. Moreover, in 2016, Hasdemir et al. also supported that intraperitoneal administration of PFD (150 mg/animal ip~2 ml of 7.5% *w*/*v*) significantly abolished adhesion scores, fibrosis, and vascular proliferation as well as the protein concentrations of IL-17 and TGF-*β*1 [30]. Intriguingly, in the cellular model of adhesion, PFD at 100 *μ*g/ml reprogrammed the IL-4/IL-13-induced M2 fibrotic macrophages and polarized towards M1 cells by decreasing the levels of TGF-*β*1, collagen type one, and related markers, including YM-1 and CD206 and transferrin receptors [41]. Collectively, these studies can support the results of the positive control PFD used in the current study.

It has been demonstrated that oxidative stress is one of the major factors responsible for adhesion development. Activated oxygen and nitrogen species stimulate fibroblastic cells' growth in damaged areas and lead to fibrosis formation [51, 52]. Therefore, we investigated MDA levels as an oxidative agent and GSH as antioxidative factors. We found that *C. sativus* extract (0.25-0.5% *w*/*v*~25 and 50 mg/kg) meaningfully reduces MDA level and enhances GSH level following postoperational-induced peritoneal adhesion in a concentration-dependent manner. In line with our results, Ghadrdoost et al. determined that *C. sativus* extract (30 mg/kg) and crocin (15 and 30 mg/kg) diminish lipid peroxidation by reducing the MDA level. Simultaneously, the extract and its active constituent augmented total antioxidant activity, glutathione peroxidase, glutathione reductase, and superoxide dismutase activities following the oxidative stress and spatial learning and memory deficits induced by chronic stress in rats [53].

Additionally, it has been demonstrated that *C. sativus* aqueous extract (10, 20, and 40 mg/kg) mitigated MDA and nitric oxide levels, while it appended the levels of GSH and catalase and SOD activities following streptozotocin-induced diabetes in rats [54]. Akbari and coworkers figured out that *C. sativus* extract (40 mg/kg) attenuates MDA and IL-6 levels and propagates GSH level as well as glutathione peroxidase activity in exercised rats [55]. In one study, *C. sativus* stigmas and high-quality byproducts (petals+anthers-CTA) extracts (25 *μ*g/ml) provided a significant decrement in ROS and lactate dehydrogenase levels in human colon cancer (HCT116) cell lines following hydrogen peroxide-induced oxidative stress. Moreover, CST and CTA alleviated MDA levels in rat colon specimens challenged with *E. coli* lipopolysaccharide [56]. Crocin, one of the major active constituents of *C. sativus*, decreased MDA and xanthine oxidase while it increased GSH levels in streptozotocin-induced diabetic rats [57]. These studies may endorse our results regarding the antioxidant effects of *C. sativus* extract.

Inflammation and inflammatory cytokines are considered one of the most critical factors responsible for postoperative adhesion formation. In damaged tissue, macrophages secret IL-6 and TNF-*α*, which cause coagulation and the formation of fibrin layers that extend adhesion [3]. By contrast, IL-10 as an anti-inflammatory cytokine inhibits the secretion of pro-inflammatory cytokines, such as IL-8, IL-6, and TNF-*α*, and plasminogen activator enzymes and prevents tissue damage [53]. Therefore, we measured the effects of *C. sativus* on the levels of TNF-*α*, IL-6, IFN-*γ*, and PGE_2_ as inflammatory cytokines and IL-4 and IL-10 concentrations as anti-inflammatory cytokines. Our results revealed that *C. sativus* extract (0.25-0.5% *w*/*v*) concentration-dependently reduces the levels of TNF-*α*, IFN-*γ*, PGE_2_, IL-6, and IL-4, while making a significant increment in IL-10 level following postoperational-induced adhesion in the rat. In line with our animal results, we observed that the level of IL-10 was increased following the TGF-*β*1 stimulation in the macrophage cell line. However, the level of TNF-*α* as an inflammatory cytokine was propagated at higher concentrations of the extract. In fact, this phenomenon was in contrast to the anti-inflammatory effects of the *C. sativus* extract observed in the animal section. It can be justified that TGF-*β*1 slightly reduces the TNF-*α* and leads to provide fibrotic macrophages (M2 cells), which produce higher levels of fibrotic and angiogenesis factors, as shown in our results of Figures 9 and 10. Indeed, by TGF-*β*1 stimulation, the macrophage phenotypes were polarized towards M2 cells by decreasing the level of increasing the level of Arg-1 as a marker of M2 cells and iNOS as a marker of M1 macrophage cells and iNOS/Arg-1 ratio (M1/M2 ratio). It justifies that the extract provides no inflammatory state but modulates the macrophage polarization towards nonfibrotic phenotypes that secrets higher TNF-*α* levels. Moreover, we assessed the level of IL-10 as supportive data, which endorse our vision on the direct effects of the extract on macrophage polarization and increasing the TNF-*α* level.

Christodoulou et al. demonstrated that *Crocus sativus* L. aqueous extract (30, 60, and 90 mg/kg/day) reduces IL-6, TNF-*α*, monocyte chemoattractant protein-1, matrix metalloproteinase- (MMP-) 2, MMP-3, and MMP-9 levels, and MMP/TIMP-2 ratio in diabetic atherosclerotic C57BL/6J wild-type mice [58]. In another study, *Crocus sativus* (20, 40, and 80 mg/kg) diminished IL-4 and NO levels, while it enhanced IFN-*γ* and IFN-*γ*/IL-4 ratio levels in ovalbumin-sensitised guinea pigs [59]. Faridi and coworkers suggested that hydroalcoholic extract of *C. sativus* (500 mg/kg) mitigates IFN-*γ* and IL-17 and augments IL-10 levels following streptozocin-induced autoimmune diabetes in C57BL/6 mice [60]. However, the levels of the extract were considerably higher than what we investigated in our study. Additionally, Hemshekhar et al. reported that crocin (10 and 20 mg/kg), one of the major active constituents of *C. sativus*, alleviates MMP-13, MMP-3, MMP-9, TNF-*α*, IL-1*β*, NF-*κ*B, IL-6, COX-2, PGE_2_, and ROS levels following Freund's complete adjuvant- (FCA-) induced arthritis in rats [61]. In another study, crocin (100 and 200 ppm~1 and 2% *w*/*v*) made a significant decrement in the levels of mRNA expression of TNF-*α*, IL-1*β*, IL-6, IFN-*γ*, NF-*κ*B, COX-2, and iNOS and propagated Nrf2 mRNA expression in the colorectal mucosa following dextran sodium sulfate-induced colitis [62]. These studies may support our results regarding the anti-inflammatory properties of *C. sativus* extract.

The previous human and animal studies indicated that the levels of TGF-*β* are significantly increased in the peritoneal adhesions [2, 24, 50]. TGF-*β* is a suppressive and fibrotic cytokine that controls reproduction, differentiation, cell apoptosis, and wound healing. The active form of TGF-*β* increases the secretion of the extracellular matrix, leading to the formation of adhesion [3, 63]. Vascular endothelial growth factor (VEGF) is another growth factor and potent mitogen for endothelial cells and a vital angiogenesis factor, which is essential for wound healing and adhesion formation [2, 24, 50]. In fact, VEGF production is stimulated by lactate in macrophages, and lactate accumulation plays a critical role in adhesion development [2, 3, 24, 50]. It has been emphasised that the anti-VEGF monoclonal antibody decreases the postoperational peritoneal adhesion in mice [64]. The current study results figured out that *C. sativus* extract (0.25-0.5% *w*/*v* ~25 and 50 mg/kg) provided a significant and concentration-dependent decrement in TGF-*β* and VEGF levels following the postoperational peritoneal adhesion. Interestingly, our in vitro study found that VEGF level was also meaningfully abrogated by *C. sativus* extract in a concentration-dependent manner.

In line with our results, Alemzadeh and Oryan investigated that *C. sativus* extract (20% *w*/*w*; topically) diminishes the expression of IL-1*β* and TGF-*β*1 and improves wound healing following the burn wounds in rats [65]. Additionally, crocin (20 mg/kg) mitigated TGF-*β*, NF-*κ*B, and IL-6 expression levels following streptozocin-induced diabetic nephropathy in rats [66]. Algandaby also showed that crocin (25 and 100 mg/kg) attenuates the expression of TGF-*β*, alpha-smooth muscle actin (*α*-SMA) and collagen 1-*α*, NF-*κ*B, COX-2, IL-1*β*, and TNF-*α* following thioacetamide-induced liver fibrosis in mice [67]. Kermani and coworkers demonstrated that *C. sativus* (100 mg/day) reduces VEGF, IL-2, and IL-1*β* while enhancing IL-10 levels compared to the placebo group in metabolic syndrome patients [68]. Furthermore, *C. sativus* aqueous extract (400 and 800 *μ*g/ml) attenuated the expression levels of VEGF-A and VEGF-2 in the MCF-7 cell line and prevented angiogenesis [69]. Additionally, crocin (25 mg/kg) mitigated VEGF, IL-6, IFN-*γ*, and TNF-*α* levels in a mouse model of endometriosis [70]. In another study, crocin (250 and 500 *μ*g/kg) attenuated VEGF, MMP-2, and MMP-9 expressions and TNF-*α* and IL-6 levels while it elevated IL-10 level in melanoma metastatic model in C57BL/6 mice [71]. These studies may endorse our animal and cellular results regarding the antifibrotic and antiangiogenesis effects of the extract.

Tissue plasminogen activator (tPA) is classified as a serine protease that prevents the progression of mesothelial cell adhesion by inhibiting plasminogen transformation to plasmin. In low tPA level condition, fibrin masses form a clot attacked by fibroblasts, collagens, and other proteins that lead to adhesion formation [72]. Plasminogen activator inhibitor (PAI), which is present in plasma, inhibits the tPA. Increasing the PAI level and decreasing the tPA level and tPA/PAI ratio are considered adhesion development causes [3, 73]. In one study, Atta and coworkers found lower TGF-*β*1 and PAI and higher tPA levels in the group with a lower rate of postoperative adhesion formation in rats [74]. Therefore, we determined the levels of TPA, PAI and the ratio of TPA/PAI. We found that *C. sativus* (0.25-0.5% *w*/*v*~25 and 50 mg/kg) mitigates PAI level and propagates tPA and TPA/PAI ratio levels in a concentration-dependent manner following the postoperational induced peritoneal adhesion. Tsantarliotou and coworkers suggested that crocin at both low and high doses (10 and 100 mg/kg) could diminish PAI-1 levels in the liver and brain tissue following lipopolysaccharide-induced thrombosis in rats [75].

## 5. Conclusion

**Figure 11:**
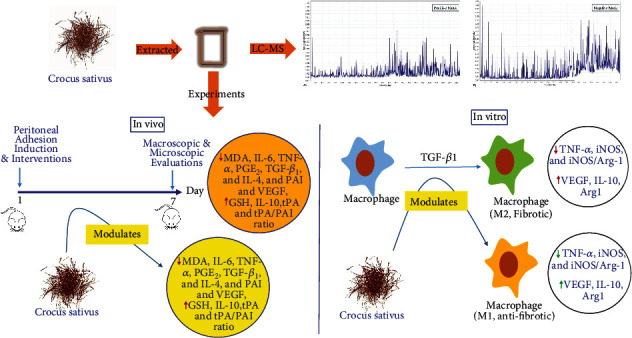
Preventive effects of *C. sativus* extract against postsurgical-induced peritoneal adhesion.

In summary, our results revealed that *C. sativus* could prevent postoperative peritoneal adhesion through attenuating adhesion score, oxidative stress, inflammatory cytokines, fibrosis, and angiogenesis markers, while propagating antioxidant and anti-inflammatory markers and tPA (Figure 11). Moreover, the current study indicated that *C. sativus* reduces peritoneal adhesion formation by modulating the macrophage polarization from M2 towards M1 cells (Figure 11). It could be concluded that *C. sativus* may be the right candidate for preventing postoperative peritoneal adhesion.

## Figures and Tables

**Table 1:** Adhesion score categories according to Nair et al. [32].

Grades	Description of adhesive bands
0	The complete absence of adhesions
1	A single band of adhesion, between viscera or from viscera to the abdominal wall
2	Two bands, either between viscera or from viscera to the abdominal wall
3	More than two bands, between viscera or viscera to the abdominal wall or whole intestines forming a mass without being adherent to the abdominal wall
4	Viscera directly adherent to the abdominal wall, irrespective of number and extent of adhesive bands

**Table 2:** Peak assignment of metabolites in the hydroethanol extract of *C. sativus* (*Crocus sativus* L.) using LC-MS in the negative mode.

Peak no.	Compound identification	*t*_R_ (min)	[M-1] (*m*/*z*)	Ref.
1	4-(*α*-D-glucopyranosyl)-2,6,6-trimethyl-1-cyclohexene-1-carboxaldehyde (picrocrocin)	35.3	375.2	[76]
2	Crocetin di-(*β*-D-gentibiosyl) ester	37.0	975.3	[77]
3	Crocetin (*β*-D-glucosyl)-(*β*-D-neapolitanosyl) ester	40.4	975.0	[77]
4	Crocin E	40.4	489.4	[76]
5	Crocetin	37.2	327.3	[78]
6	Dimethyl crocetin	28.8	355.3	[78]
7	Quercetin 3-orutinosylrhamnoside	29.4	755.7	[77]
8	Quercetin 3-O-rutinoside	29.8	609.6	[77]
9	Isorhamnetin-3-orutinosylrhamnoside	35.8	769.5	[78]
10	Narcissin	42.1	623.8	[78]
11	Nepetalic acid	35.2	183.1	[79]
12	Geranic acid	30.6	167.0	[79]
13	Dihydrojasmonic acid, methyl ester	32.7	225.0	[79]
14	Kaempferol 3-glucoside	3.1	447.8	[77]
15	Angoluvarin	16.5	483.42	[79]
16	Isococculidine	5.1	284.6	[79]
17	4-Apiosyl-glucoside	1.1	593.3	[79]

**Table 3:** Peak assignment of metabolites in the hydroethanol extract of *C. sativus* (*Crocus sativus* L.) using LC-MS in the positive mode.

Peak no.	Compound identification	*t*_R_ (min)	[M+1] (*m*/*z*)	Ref.
1	Cinnamyl isovalerate	35.0	219.12	[79]
2	Crocin E	36.0	491.7	[78]
3	Crocetin	44.1	329.4	[78]
4	Isorhamnetin-3-O-*β*-D-glucopyranoside	38.0	479.7	[78]
5	Kaempferol 3-sophoroside-7-glucoside	42.8	772.2	[79]
6	Crocusatin	9.6	185.4	[80]
7	Taxifolin 7-O-hexoside	24.2	466.3	[80]
8	Kaempferol 3-O-hexoside-7-O-(acetyl)-hexoside	25.7	653.1	[80]
9	Sinapic acid	32.8	224.8	[80]
10	Adenosine	38.0	268.0	[80]
11	Tamarixetin 3-O-bihexoside	36.2	641.1	[80]
12	Rhamnetin	30.8	317.1	[79]
13	Naringenin	33.3	273.3	[79]
14	Tamarixetin O-kaempferol biflavonoid hexoside	23.1	747.1	[78, 80]
15	Karatavigenin B	24.3	569.3	[79]
16	Anhalonidine	28.0	224.1	[79]
17	Baicalein	30.9	271.2	[79]
18	4,6,8-Megastigmatriene	31.7	177.7	[79]

## Data Availability

The data used to support the findings of this study are available from the corresponding author upon reasonable request.

## Abbreviations

COX2: Cyclooxygenase-2
GSH: Glutathione
IFN-*γ*: Interferon-gamma
IL-10: Interleukin-10
IL-4: Interleukin-4
IL-6: Interleukin-6
iNOS: Nitric oxide synthase
MDA: Malondialdehyde
MMP: Matrix metallopeptidase
NF-*κ*B: Nuclear factor kappa-light-chain-enhancer of activated B cells
NO: Nitric oxide
PA: Plasminogen activator
PFD: Pirfenidone
PGE_2_: Prostaglandin E2
SOD: Superoxide dismutase
TAP: Tissue plasminogen activator
TGF-*β*: Transforming growth factor-beta
TIMP-2: Tissue inhibitor of metalloproteinases-2
TIMPs: The tissue inhibitors of metalloproteinases
TNF-*α*: Tumour necrosis factor-alpha
VEGF: Vascular endothelial growth factor
*α*-SMA: Alpha-smooth muscle actin.
